# A One-Step Homogeneous Sandwich Immunosensor for *Salmonella* Detection Based on Magnetic Nanoparticles (MNPs) and Quantum Dots (QDs)

**DOI:** 10.3390/ijms14048603

**Published:** 2013-04-22

**Authors:** Hua Kuang, Gang Cui, Xiujin Chen, Honghong Yin, Qianqian Yong, Liguang Xu, Chifang Peng, Libing Wang, Chuanlai Xu

**Affiliations:** 1 School of Food Science and Technology, State Key Lab of Food Science and Technology, Jiangnan University, Wuxi 214122, Jiangsu, China; E-Mails: chenxiujin9610@126.com (X.C.); yin0713@126.com (H.Y.); yongqian-q@163.com (Q.Y.); xuliguang2006@126.com (L.X.); pcf@jiangnan.edu.cn (C.P.); xcl@jiangnan.edu.cn (C.X.); 2 YanCheng Teachers University, Yancheng 214122, Jiangsu, China; E-Mail: cuigang@ycit.cn; 3 Research Center of Hunan Entry-Exit Inspection and Quarantine Bureau, Changsha 410001, Hunan, China; E-Mail: wanglb1@126.com

**Keywords:** *Salmonella*, fluorescent immunoassay, magnetic separation, quantum dots

## Abstract

Simple immuno-magnetic separation tandem fluorescent probes based on quantum dots-antibody (QDs-Ab) were developed to detect *Salmonella* with sensitivity of 500 cfu mL^−1^. With two monoclonal antibodies, which recognize different antigenic determinant on the surface of *Salmonella*, we prepared antibody-coated magnetic nanoparticles (MNPs) and conjugates of QDs-Ab. The immune-magnetic beads were verified with high enrichment efficiency for *Salmonella* (90%). A sandwich structure formed if the *Salmonella* solution was mixed together with immune-beads and QDs-Ab, and the fluorescent single from QDs was related to the amount of *Salmonella*. A linear response between fluorescence intensity and various concentrations of *Salmonella* (2.5 × 10^3^ to 1.95 × 10^8^ cfu mL^−1^) were observed with this proposed method. The total assay time for *Salmonella* was 30 min, and no cross-reaction to other microbial strains, such as *Staphylococcus aureus*, *Escherichia coli* (*E. coli*) and *Escherichia coli O157:H7* (*E. coli O157:H7*), were found using this detection system. All our results showed that the simple homogeneous immunoassay could be applied in *Salmonella* screening without time-consuming extra-enrichment of bacteria.

## 1. Introduction

*Salmonella* is a common food-borne pathogen that causes food contamination, which has resulted in higher economic losses and poses a significant threat to public health [1,2]. Salmonellosis is the most frequently reported disease worldwide, initiating into diarrhea, cramps, vomiting and fever symptoms [3]. These infections cause significant morbidity and mortality that are particularly severe in infants, the elderly and immune-compromised patients [4–6]. Annually, an estimated 33 million people in the world suffer from typhoid fever and 600,000 deaths are reported among them [7,8]. Even in developed countries, such as the United States, the outbreak incidences of *Salmonella* infections have been increasing in recent decades. The most easily contaminated foods by *Salmonella* are meat; poultry and egg products [9]. Hence, the development of *Salmonella* screening method is crucial to safeguard human health and identify their distribution for better management of food safety.

To date, some approaches for *Salmonella* detection have been developed, including conventional culture methods, immunoassays [10–17] and polymerase chain reaction (PCR) measures [18–22]. However, these measures either require a long-time pre-enrichment step or depend on instruments and professionals in laboratory.

In this paper, we present a novel immuno-sensor using quantum dots (QDs) as fluorescent label and magnetic nanoparticles (MNPs) as enrichment reagent. A pair of monoclonal antibody against *Salmonella* was used in our detection design. One antibody was conjugated to QDs for detection and the other was bound to MNPs for capturing the targeted bacteria in solution. A fluoresce single was quantitative based on the forming a sandwich structure (QDs-bacteria-MNPs).

## 2. Materials and Methods

### 2.1. Materials

All bacterial strains used in this study were purchased from the China Center of Industrial Culture Collection (Beijing, China), including *Salmonella*, *Staphylococcus aureus*, *E. coli* and *E. coli O157:H7*. The paired monoclonal antibodies, QDs and MNPs were all prepared in our lab. Two antibodies were obtained through hybridoma selection process. The affinity constants were calculated as 1.8 × 10^9^ and 2.2 × 10^9^, respectively, for these two anti- *Salmonella* antibody.

Bovine serum albumin (BSA) was obtained from Solarbio Science & Technology, Co, Ltd. (Beijing, China). *N*-hydroxysulfosuccinimide sodium salt (sulfo-NHS) was purchased from J&K Scientific, Ltd. (Beijing, China). 1-ethyl-3-(3-dimethylaminopropyl)-carbodiimide (EDC) was purchased from Aladdin Reagent (Shanghai, China). Biotin and streptavidin were purchased from Sigma-Aldrich (Shanghai, China). Other chemicals were purchased from the Shanghai Chemical Reagent Company (Shanghai, China). All water in this experiment was ultra-water from Millipore water purification system (18.2 MΩ.cm; EMD Millipore Corporation, Billerica, MA, USA).

### 2.2. Design of the Sandwich Immunoassay for *Salmonella* Detection

**Figure 1:**
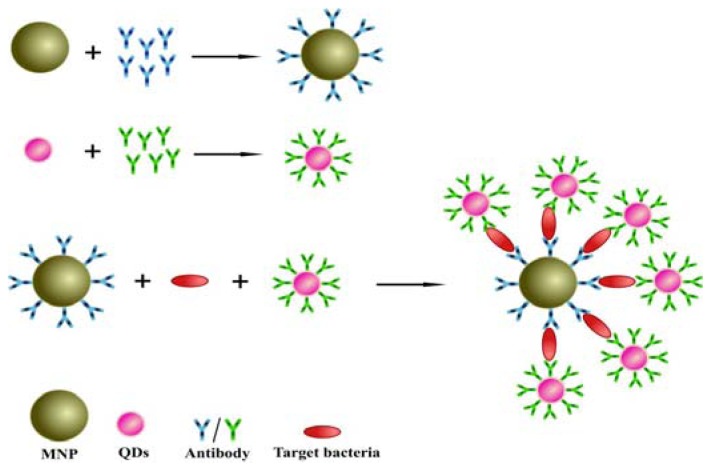
Schematic diagram of detection principle based on a sandwich assay using magnetic nanoparticles and quantum dots for *Salmonella* detection.

A schematic of the sensing principle was illustrated in Figure 1. Three critical materials, including MNPs, QDs and two anti-*Salmonella* antibodies, were used in this detection system. The anti-*Salmonella* antibodies were immobilized individually on the MNPs and QDs. The two antibodies recognize different antigenic determinants of *Salmonella*. For assay, the sample solution was sufficiently incubated with immune-beads and QD probes in a tube for a period. Then, the tube was put in a magnetic field for separating the MNPs-bacteria-QDs from solution. The isolated *Salmonella* coupled with the QDs was quantified using a fluorescence spectrophotometer (F-7000, Hitachi Ltd., Tokyo, Japan). The increasing *Salmonella* concentrations allow more QDs to be captured by magnetic beads resulting in more intensive fluorescence single.

### 2.3 Functionalization of MNPs with Antibody

The MNPs used here was modified with carboxyl group on their surface (particle size 100 nm). EDC and sulfo-NHS were applied as linkers in this experiment and they were dissolved to phosphate-buffered saline (PBS, 0.1 M, pH 7.2) with final concentrations of 0.5 and 0.2 mg mL^−1^, respectively. One milliliter of the linker solution was mixed with equal volume of MNPs and held for 15 min at room temperature, then 100 μL aliquots of anti-*Salmonella* antibody (Ab-1) was added drop-wise into the solution. The reaction was carried out at 37 °C for 2 h, and then the MNP beads were isolated using magnet. After removal of the liquid in the tube, 1% BSA solution in PBS (*g*/*v*, 1 mL) was re-dispersed on MNPs with continuous stirring for 1 h at 37 °C to block the bareness sites on the surface of MNPs. Following another magnetic separation step, the residues were dissolved in 1 mL PBS and then stored at 4 °C for further use. The capture ability of *Salmonella* from immune-magnetic beads was evaluated using the counted *Salmonella* (1.16 × 10^5^ cfu mL^−1^). After half an hour’s incubation, the immune-magnetic beads were recovered using magnetic separation.

The separated MNPs were washed 5 times and re-suspended in nutrient broth. Then, plate counts were performed to evaluate the enrichment efficiency of immune-magnetic beads.

#### 2.3.1. Synthesis of QDs-Ab Conjugates

The anti-*Salmonella* antibody (Ab-2) and QDs were coupled by a biotin-streptavidin bridge. The conjugation method is the same as our previous reports [23,24]. The prepared QDs-Ab conjugates were dissolved in PBS (200 μL) and stored at 4 °C for the following use.

#### 2.3.2. *Salmonella* Sample Analysis

The sample solution (1 mL) was added with 50 μL of immune-MNPs beads, and following this, 50 μL of QDs-Ab conjugates were added. The mixture solution was allowed for 30 min incubation forming the sandwich structure of immune-MNPs/*Salmonella*/QDs-Ab. With a magnetic separation, the residual QDs were removed from the solution. The *Salmonella* cells bound to the QDs and MNPs were re-suspended in 1 mL PBS for fluorescent measurement. Various concentrations (from 10^2^ to 10^8^ cfu/mL) of *Salmonella* in PBS buffer were tested with this proposed detection system. The values of the fluorescence intensity were plotted against the *Salmonella* to produce a linear curve. The specificity of this proposed immunoassay was tested with other bacteria, including *Staphylococcus aureus*, *E. coli* and *E. coli O157:H7*.

## 3. Results and Discussion

### 3.1. Characterization of QD-Ab

**Figure 2:**
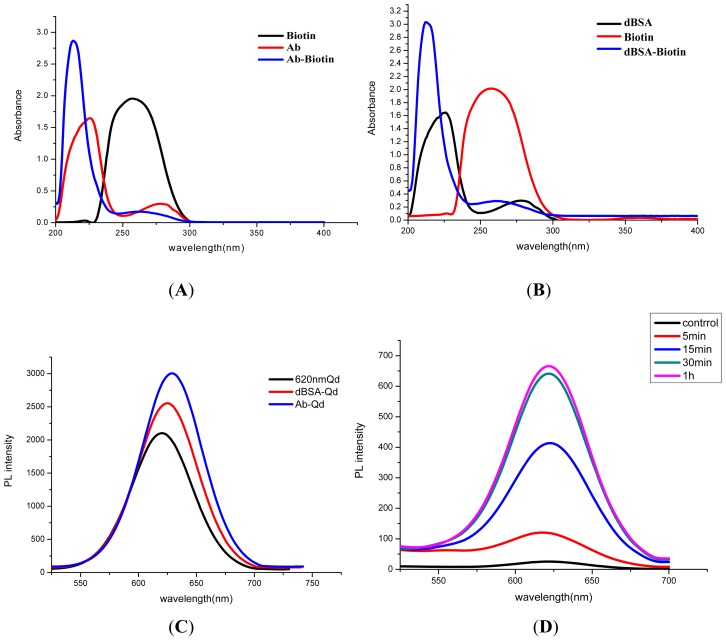
Characterization of conjugates and optimization of the incubation time for assay system. (**A**) The UV spectra for biotin-antibody conjugates; (**B**) the functioned biotin with dBSA; (**C**) fluoresce tests for conjugates of quantum dot (QD) and antibody; (**D**) optimization of incubation time for immune-MNPs, QDs-antibody (Ab) and *Salmonella*.

Both antibody and QD were functioned using biotin, and then two parts were bridged using streptavidin through the specific affinity reaction. The successful preparation of Ab-biotin complex was verified with UV detection. As it was shown in Figure 2A, the characteristic absorption peak of antibody was located at 215 nm and 278 nm, and the peak for biotin was at 260 nm. The product of biotin-Ab exhibited both typical absorption peaks of biotin and antibody, respectively, at 260 nm and 215 nm. The modification of biotin on the QD was assisted with dBSA, which was prepared with NaBH_4_ reduction beforehand [23]. The UV spectra in Figure 2B confirmed the coupling between dBSA and biotin.

With the connecting agent of streptavidin, the antibody was successfully anchored on the surface of QDs. The original QD showed the typical fluorescence emission peak at 620 nm, while an obvious red shift of the conjugates of QD-dBSA-biotin (QD-dBSA, 625 nm) and QD-Ab (630 nm) were seen in Figure 2C.

### 3.2. Optimization of the Immunoassay System

With gradient dilutions and then plate counts, the absorbed *Salmonella* by immune beads was calculated as (1.05 ± 0.12) × 10^5^ out of the total amount of 1.15 × 10^5^ cfu mL^−1^, resulting in the average 90% binding efficiency.

In evaluation of the optimum incubation period for sample solution, immune-beads and QD probes in the assay buffer, various incubation times (5, 15, 30 and 60 min) were tested. The total amount of *Salmonella* was 1.16 × 10^5^ cfu for optimization tests, and a blank sample (not containing *Salmonella*) was used as a control. The photoluminescence (PL) intensity from the formed sandwich products was determined. The results from Figure 2D indicated that the fluorescence intensity was enhanced with increasing incubation time from 5 to 30 min. However, no significant differences were observed if the incubation was raised to 60 min. Hence, the optimal incubation time was 30 min, which reflected the high affinity among immune-beads, QD probes and *Salmonella*.

### 3.3. Performance Assessment of *Salmonella* Detection

**Figure 3:**
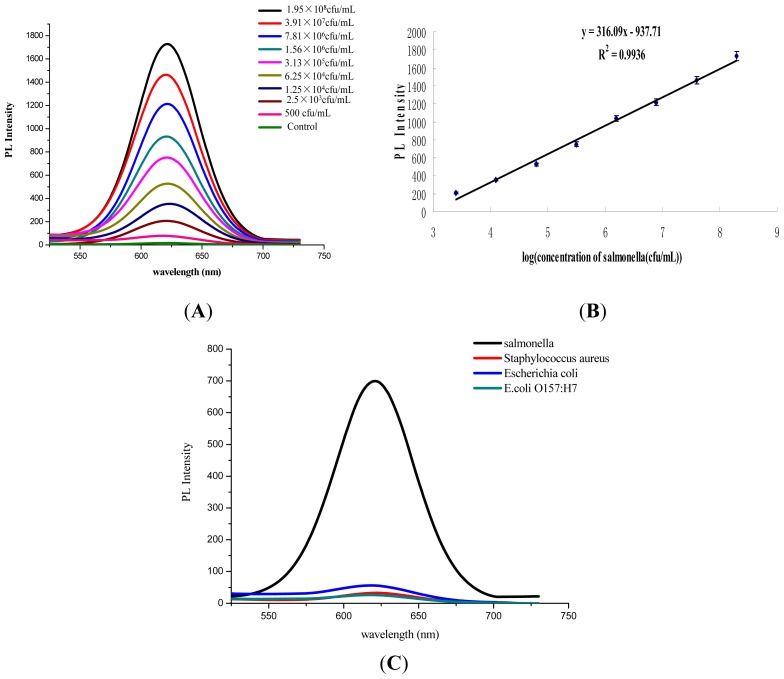
Performance evaluation of the homogeneous immunoassay for *Salmonella*. (**A**) Fluorescence emission spectra for detection of various concentrations of *Salmonella*; (**B**) the plotted linear curve based on the various concentrations of *Salmonella*. Error bar represents standard deviation of five measurements; (**C**) cross-reaction determination for other bacteria.

The assay sensitivity was evaluated using a series of *Salmonella* concentrations (500 to 1.95 × 10^8^ cfu mL^−1^). As shown in Figure 3A, the photoluminescence (PL) intensity rose gradually with the increasing concentration of *Salmonella*. It demonstrated a good linear relationship when the bacteria concentration ranging from 2.5 × 10^3^ to 1.95 × 10^8^ cfu mL^−1^ with a correlation coefficient of 0.9936 (Figure 3B). A test sample was considered positive if the ratio (P/N) of the optical density (OD) value in the test well to that of the negative control well was ≥2.1. The sensitivity of this proposed assay was 500 cfu mL^−1^.

In specificity tests, the control sample (no containing any bacteria) and three pathogens, including *Staphylococcus aureus*, *E. coli* and *E. coli O157:H7*, were detected. All tested bacteria were at the 10^5^ level. As it was shown in Figure 3C, very slight PL intensity (<80 au) could be detected for the control solution and non-targeted bacteria, while a strong fluorescence signal was observed for *Salmonella* (~700 au). All the results indicated that the immune-MNPs tandem QDs-Ab system was very specific in *Salmonella* detection.

## 4. Conclusions

A sensitive and simple immunoassay was developed for *Salmonella* here. With the efficient enrichment of immune-MNPs, the sample could be completed in half an hour. Due to the predominant fluorescent character of QDs, quantitative analysis was possible for *Salmonella* with a linear curve. Without complex pre-culturing procedures, the proposed method could be directly detected samples with the sensitivity of 500 cfu mL^−1^ for *Salmonella.* This immunoassay was simple and fast, which makes it a great potential for being applied in surveillance of *Salmonella* contamination.
